# A plant virus differentially alters DNA methylation in two cryptic species of a hemipteran vector

**DOI:** 10.1038/s44298-024-00044-2

**Published:** 2024-08-12

**Authors:** Michael A. Catto, Saptarshi Ghosh, Sudeep Pandey, Banani Mondal, Alvin Simmons, Brendan G. Hunt, Rajagopalbabu Srinivasan

**Affiliations:** 1 Department of Entomology, University of Georgia, Griffin, GA USA; 2 U.S. Department of Agriculture, Charleston, SC USA

**Keywords:** Computational biology and bioinformatics, Genetics

## Abstract

Epigenetic patterns including DNA methylation are known to vary between distantly related species, but it is not clear how these patterns differ at an intraspecific level. The sweetpotato whitefly, *Bemisia tabaci* (Gennadius) (Aleyrodidae; Hemiptera), encompasses several cryptic species. These cryptic species possess highly similar genomes but exhibit substantial biological and physiological differences. *B. tabaci* cryptic species are invasive, highly polyphagous, and transmit an array of plant infecting single stranded DNA viruses (ssDNA) –begomoviruses. In this study, DNA methylation patterns around genes and genomic features of two prominent *B. tabaci* cryptic species were investigated following acquisition of a monopartite ssDNA virus –tomato yellow curl virus. The cryptic species investigated included: B (also known as Middle East Asia Minor 1) and Q (also known as Mediterranean). Genomic features, such as promoters, gene bodies, and transposable elements were assessed for methylation levels in both B and Q cryptic species. Despite overall similar trends, both cryptic species showed differences in methylation levels between these genomic features. Virus induced differentially methylated regions were associated with predominantly distinct genes in B and Q cryptic species. All differentially methylated regions were assessed for differential gene expression and alternative splicing events with and without virus acquisition. DNA methylation levels were found to have a negative correlation with differential gene expression in both B and Q cryptic species. The differentially expressed genes were further grouped into hyper- and hypomethylated clusters. These clusters included genes with implications for virus-vector interactions including immune functions and xenobiotics’ detoxification. The observed DNA methylation pattern differences within each cryptic species could, in part, explain some of the biological and physiological differences between them.

## Introduction

Epigenetic mechanisms play an important role in the phenotypic diversity and adaptation of eukaryotes to their biotic environments, including interactions with microbes. Among those mechanisms, DNA methylation has emerged as a widely studied epigenetic modification^1–4^. These epigenetic modifications are regulated by enzymes known as DNA methyltransferases (DNMTs)^5^. DNA methylation in eukaryotes alters transcription factor binding and thus directly or indirectly contributes to variation in gene expression, alternative splicing (AS) events, and transposable element (TE) silencing^6–10^. In the vertebrate genomes, DNA methylation is globally targeted, present in many gene promoters, and enriched in transposable elements^11^. However, there is currently no robust evidence among insects for DNA methylation enrichment in TEs or in promoters^12^. Gene regulation in insects is believed to be mediated primarily by intragenic DNA methylation, which in turn is known to influence AS and reduce transcriptional noise^10,13^. Comparison of major insect groups revealed that DNA methylation occurs in a larger proportion of the genomes of hemimetabolous insect orders such as Hemiptera than in holometabolous insect orders^1,14^. Global methylation patterns following interactions with pathogens also may vary across insect orders and may relate to the evolution of insect immune responses^15,16^.

The sweetpotato whitefly, *Bemisia tabaci* (Gennadius) (family Aleyrodidae; order Hemiptera), is an agricultural pest with global importance and serves as a vector for over 300 diverse plant pathogenic viruses including single stranded DNA (ssDNA) viruses belonging to the genus *Begomovirus*^17^. Begomoviruses cause significant losses to global food production^18,19^. These viruses have high mutation rates and exist as quasispecies with variant genomes within a population^20^. For this investigation, a common monopartite Begomovirus *viz*., tomato yellow leaf curl virus (TYLCV) was chosen. TYLCV is transmitted by *B. tabaci* in a persistent-circulative, non-propogative manner^21–23^. Viruses transmitted in this manner are typically acquired by phloem-feeding insects within several hours of feeding, and the acquired viruses persists within insects for life^24–27^. The virus upon acquisition traverses between membranes at the midgut into the hemolymph and then from the hemolymph into the salivary glands via highly specific receptor-mediated endocytosis^28,29^. The viruses that traverse into the salivary glands can then be reinoculated into susceptible plants via feeding^28,29^. There also is a latent period between virus acquisition and inoculation^24–27^. The process of TYLCV transmission involves several complex interactions between the virus and whitefly proteins^30^, and it is not clear if these complex interactions would have any effect on DNA methylation.

*B. tabaci* species complex comprises 5 to 34 different cryptic species with each exhibiting unique biological and physiological characteristics^31–35^. Notable members of this species complex include the B cryptic species, which also is referred to as Middle East Asia Minor 1, and the Q cryptic species, which also is referred to as Mediterranean (MED)^36,37^. Both B and Q, as well as the Sub-Saharan Africa-East and Central Africa (SSA-ECA) cryptic species have genomic resources available^38–43^. B and Q cryptic species were selected for comparative analysis in this study, as they are both present in the United States^24,44–46^. The B cryptic species is widespread, whereas Q cryptic species is a relatively recent introduction into the United States^46,47^. Both B and Q cryptic species efficiently transmit TYLCV; however, the B cryptic species has been observed to transmit a wider array of begomoviruses than the Q cryptic species^48^.

Differences in virus transmission capabilities between the cryptic species, as consequences of differential interactions of cryptic species with begomoviruses^49^, could variably impact their DNA methylation profiles and defenses against the virus. The global and gene level DNA methylation patterns were profiled in response to virus infection in B and Q cryptic species to shed light on potential evolutionary variations in gene regulation between the cryptic species of *B. tabaci*. DNA methylation patterns within whitefly TEs were investigated to contribute to further describing the genetic landscape of *B. tabaci*^50–53^. The relationship between differential methylation, differential expression, and AS was assessed following virus acquisition^27^. Protein clusters (gene families) were characterized to assess the functions associated with virus response related to differential methylation and differential expression. Several candidate protein clusters related to phytovirus acquisition and retention were identified.

## Results

### DNA methylation in proximity to genomic features

**Fig. 1:**
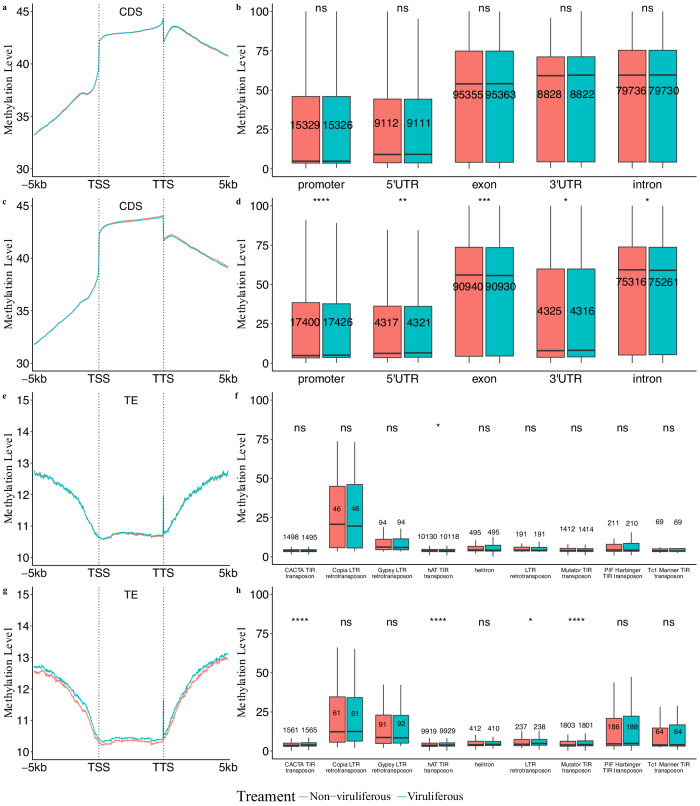
CpG methylation levels of genomic features within non-viruliferous and viruliferous *Bemisia tabaci.* B cryptic species methylation patterns within (**a**) the coding sequences (CDS) and 5 kb upstream and downstream of the translation start site (TSS) and translation termination site (TTS), respectively, and (**b**) the promoters, 5’-UTRs, exons, introns, and 3’-UTRs. Q cryptic species methylation pattern within (**c**) the CDS and 5 kb upstream and downstream of the TSS and TTS, respectively, and (**d**) the promoters, 5’-UTRs, exons, introns, and 3’-UTRs. B cryptic species methylation patterns within (**e**) the transposable elements (TEs) and 5 kb upstream and downstream of the TSS and TTS, respectively, and (**f**) various transposable element superfamilies. Q cryptic species methylation pattern within (**g**) the TEs and 5 kb upstream and downstream of the TSS and TTS, respectively, and (**h**) various transposable element superfamilies. Comparisons between non-viruliferous and viruliferous groups were conducted via Wilcoxon signed-rank tests. Statistical significance levels are indicated by asterisks in the figure: ns (not significant) for *p* > 0.05, * for *p* ≤ 0.05, ** for *p* ≤ 0.01, *** for *p* ≤ 0.001, and **** for *p* ≤ 0.0001.

Global DNA methylation patterns were assessed within the *B. tabaci* B and Q cryptic species genomes (~650 Mb), which had assemblies with Benchmarking Universal Single Copy Orthologs (BUSCO) scores of 94.6% and 93.4% completeness, respectively^38,41,54,55^. To explore the DNA methylation landscape in these whitefly cryptic species, this study analyzed the global average methylation levels and the percentage of 5-methylcytosine (5mC) in the CpG (Cytosine-phosphate-Guanine) context within the coding DNA sequences (CDS) of their genomes. Global DNA methylation levels were comparable between both B and Q cryptic species. The non-viruliferous (*n* = 5 for both cryptic species) and viruliferous (*n* = 5 for both cryptic species) groups showed relative consistency of global DNA methylation patterns in both whitefly cryptic species (Additional file 1: Fig. S1). Spearman’s rank correlation coefficient (rho) computations were found to be ~0.40 and ~0.60 in B and Q cryptic species, respectively, from all pairwise comparisons of CpG sites of non-viruliferous and viruliferous samples, indicating overall global similarities in DNA methylation (Additional file 1: Fig. S2 & S3). In both cryptic species, expected methylation patterns were observed around the CDS and within genomic features such as promoters, 5’- untranslated regions (UTRs), exons, 3’-UTRs, and introns (Fig. 1a–d; Additional file 2: Tables S1–S6; Additional file 3: Tables S7–S12). Kolmogorov-Smirnov tests were statistically significant with respect to methylation levels of the genomic features, indicating that the data were not normally distributed (B cryptic species *D* = 0.985, *P* < 2.2e^−16^; Q cryptic species *D* = 0.970, *P* < 2.2e^−16^).The promoter regions as well as the 5’-UTR had low methylation levels compared with exon and intron regions. Pairwise statistically significant differences, via Wilcoxon signed-rank tests, were observed between all genomic features in both cryptic species (Additional file 1: Table S13) and between non-viruliferous and viruliferous groups within genomic features in the Q cryptic species (Fig. 1d). Using Wilcoxon signed-rank tests, the Q cryptic species was found to exhibit slightly, but significantly lower levels of CDS, promoter, 5’-UTR, and 3’-UTR methylation compared with the B cryptic species (*P* < 2.2e^−16^).

DNA methylation was next considered in proximity to TEs in *B. tabaci*. TE counts associated with both cryptic species were enumerated (Additional file 1: Table S14). The global TE methylation patterns were below the levels observed in flanking regions, indicating that TEs were depleted for DNA methylation overall (Fig. 1e–h; Additional file 4: Tables S15 and S16). Kolmogorov-Smirnov tests were statistically significant with respect to methylation levels of the TEs, indicating the data were not normally distributed (B cryptic species *D* = 0.977, *P* < 2.2e^−16^; Q cryptic species *D* = 0.949, *P* < 2.2e^−16^). The TE methylation levels were found to be similar between the two cryptic species. Nevertheless, variations were observed between TE types, for example, the *copia* retrotransposons were the most highly methylated superfamily of TEs (Fig. 1f, h). Other superfamilies, such as *gypsy* retrotransposons, also were found to be methylated above background (Fig. 1f, h). Using pairwise Wilcoxon signed-rank tests, the methylation levels of c*opia* and *gypsy* retrotransposons were found to have a statistically significant difference in comparison with all other TE superfamilies in both cryptic species (Additional file 1: Tables S17). However, there were no statistically significant differences between methylation levels of non-viruliferous and viruliferous groups in *copia* or *gypsy* retrotransposons (Fig. 1f, h).

**Fig. 2:**
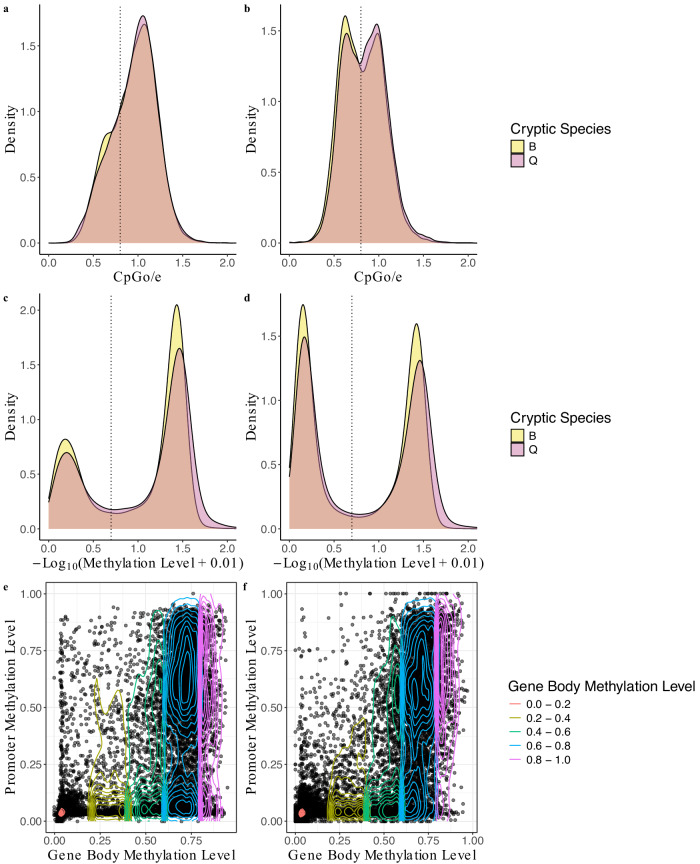
Distributions of CpGo/e and methylation levels within the promoter regions and gene bodies of *Bemisia tabaci* B (# of genes = 15,229) and Q (# of genes = 17,416) cryptic species. **a** The CpGo/e comparison reveals for B and Q, 4190 and 4636 promoters, respectively, were below 0.8 (indicated by the dotted line). **b** The CpGo/e comparison reveals that for B and Q cryptic species, 7654 and 7816 gene bodies, respectively, were below 0.8 (indicated by the dotted line). **c** The methylation level analysis shows that for B and Q cryptic species, 5059 and 5353 promoters, respectively, exceeded 20% (marked by the dotted line). **d** The methylation level analysis shows that for B and Q cryptic species, 7580 and 8240 gene bodies, respectively, exceeded 20% (marked by the dotted line). Contour density plots showing the relationship between gene body methylation levels (x-axis) and promoter methylation levels (y-axis) of (**e**) B and (**f**) Q cryptic species. Contour density lines show gene body methylation levels represented by different line types, categorized into distinct, non-overlapping intervals: 0.0–0.2, 0.2–0.4, 0.4–0.6, 0.6–0.8, and 0.8–1.0.

The methylation patterns were compared with the CpG observed-to-expected (CpGo/e) ratio, which assesses the relative abundance of CpG sites^56^. Regions with low CpGo/e may indicate that a given site could potentially be DNA methylated. Bimodality in DNA methylation and CpGo/e ratios in the promoter regions was noticed in both cryptic species (Fig. 2a, c) and gene bodies (Fig. 2b, d; Additional file 5: Tables S18 and S19). The comparison of promoter and gene body methylation levels revealed clusters at varying ranges (Fig. 2e, f). Promoter and gene body methylation levels were found to be positively correlated with one another in both cryptic species (B cryptic species Spearman’s rho = 0.545, *P* < 2.2e^−16^; Q cryptic species Spearman’s rho = 0.676, *P* < 2.2e^−16^). Additionally, CpGo/e and methylation levels in both the promoter and gene body were found to be positively correlated in both cryptic species (Additional file 1: Fig. S4a–d).

### Identification of differentially methylated regions in response to virus acquisition

Differentially methylated regions (DMRs) were investigated in response to virus acquisition in *B. tabaci* B and Q cryptic species using a 1 kb sliding window approach. Several potential candidate sites with significant differences in methylation were detected (Additional file 6: Tables S20 and S21). Genes within 1.5 kb of a DMR were classified as differentially methylated. In B cryptic species, 57 genes were identified with DMRs (Additional file 6: Table S22; Top 20 genes can be found in Table 1). Similarly, in Q cryptic species, 58 genes were identified with DMRs (Additional file 6: Table S23; Top 20 genes can be found in Table 2).

**Table 1:** Top 20 classified genes with differentially methylated regions (DMRs) in the *Bemisia tabaci* B genome within 1.5 kb proximity

ID	Description	DMR
Bta07458	Mitochondrial import inner membrane translocase subunit Tim9	16.5
Bta02188	Phosphatidylinositide phosphatase SAC1-B	−18.93
Bta10274	Pre-mRNA branch site p14-like protein	28.37
Bta03061	DNA Pol B2 domain-containing protein	–14.11
Bta00119	Tyrosine-protein kinase	8.24
Bta05233	ATP dependent DNA helicase	−14.32
Bta15720	U3 small nucleolar RNA-interacting protein 2	9.51
Bta10419	39 S ribosomal protein L21, mitochondrial	−10.66
Bta00269	FLYWCH and MULE domain containing protein	11.16
Bta15720	U3 small nucleolar RNA-interacting protein 2	−6
Bta02788	Guanine nucleotide exchange factor MSS4 homolog	−8.43
Bta03826	RNA-directed DNA polymerase from mobile element jockey	9.15
Bta03076	Reverse transcriptase	4.93
Bta06817	Protein SDE2 homolog	15.95
Bta01168	Alpha-1,3-mannosyltransferase ALG2	15.06
Bta03757	Brinker	−9.78
Bta15146	KRAB-A domain-containing protein 2	10.17
Bta00846	Endonuclease-reverse transcriptase	4.54
Bta00401	BET1-like protein	−19.74
Bta03315	Adenosine deaminase	4.51

The DMR shows the percent methylation difference between non-viruliferous and viruliferous groups.

^*^Differences were estimated at (*p* < 0.05).

**Table 2:** Top 20 classified genes with differentially methylated regions (DMRs) in the *Bemisia tabaci* Q genome within 1.5 kb proximity

ID	Description	DMR
BTA029404.1	transmembrane protein 170A	12.81
BTA021568.2	alpha-L-iduronidase isoform X1	−10.89
BTA018500.1	40S ribosomal protein S28	−15.60
BTA006475.1	FLJ37770-like protein	−4.52
BTA025943.1	gag-pol polyprotein	−7.62
BTA026677.3	39S ribosomal protein L39, mitochondrial	−14.17
BTA012310.1	transmembrane emp24 domain-containing protein 2	6.55
BTA010450.1	RNA guanine-N7 methyltransferase activating subunit	−8.62
BTA016517.1	nucleic-acid-binding protein from mobile element jockey-like	8.38
BTA020202.1	UPF0160 protein MYG1, mitochondrial isoform X2	−17.47
BTA025943.1	gag-pol polyprotein	5.09
BTA023153.1	shematrin-like protein 1	11.88
BTA029167.1	cytoplasmic FMR1-interacting protein-like isoform X1	−10.42
BTA011257.1	N-alpha-acetyltransferase 25, NatB auxiliary subunit-like	−9.66
BTA024431.1	RNA-directed DNA polymerase from mobile element jockey-like	−5.99
BTA025360.1	choline dehydrogenase 7	−7.78
BTA026680.1	NADH dehydrogenase [ubiquinone] 1 alpha subcomplex assembly factor 2 isoform X1	12.50
BTA024661.1	zinc finger protein 77 isoform X1	6.07
BTA029508.1	gag-pol polyprotein	−8.13
BTA029329.1	ras-related protein Rap-2c	−16.47

The DMR shows the percent methylation difference between non-viruliferous and viruliferous groups.

^*^Differences were estimated at (*p* < 0.05).

### Comparison of DNA methylation, gene expression, and alternative splicing in relation to virus acquisition

**Fig. 3:**
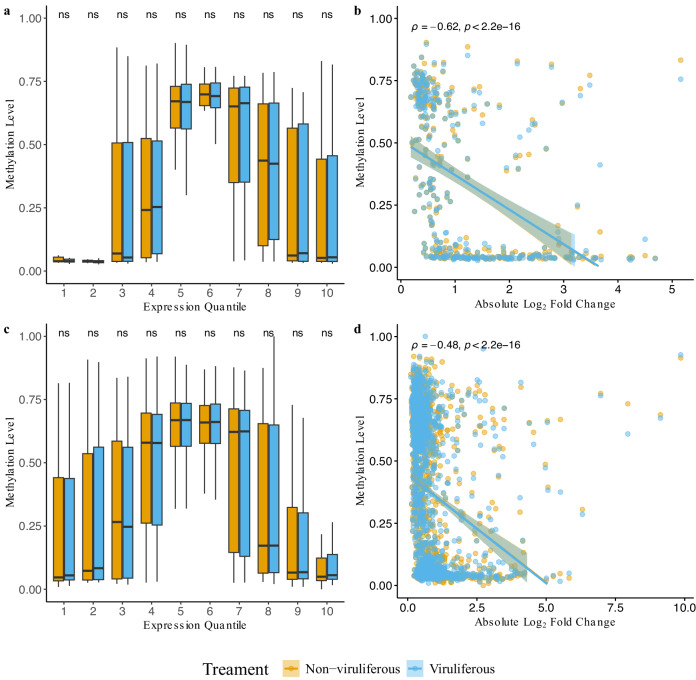
Methylation patterns related to transcript derived expression from non-viruliferous and viruliferous adult *Bemisia tabaci.* **a** Expression quantiles in relation to methylation levels in B cryptic species. **b** The absolute Log_2_ Fold Change (LFC) of differential expression in relation to methylation levels in B cryptic species. **c** Expression quantiles in relation to methylation levels in Q cryptic species. **d** The absolute LFC of differential expression in relation to methylation levels in Q cryptic species. Comparisons between non-viruliferous and viruliferous groups were conducted via Wilcoxon signed-rank tests. Statistical significance levels are indicated by asterisks in the figure: ns (not significant) for *p* > 0.05, * for *p* ≤ 0.05, ** for *p* ≤ 0.01, *** for *p* ≤ 0.001, and **** for *p* ≤ 0.0001.

This study focused on determining the methylation levels concerning virus acquisition in *B. tabaci* B and Q cryptic species. In general, higher DNA methylation levels were found to be associated with lower differential expression^45^. The expression analysis in B cryptic species identified a total of 328 differentially expressed genes (DEGs) (Additional file 7: Table S24), and in Q cryptic species, a total of 1616 DEGs were identified (Additional file 7: Table S25). Both B and Q cryptic species showed a non-monotonic association between DNA methylation and gene expression quantiles (Fig. 3a, c). Kruskal-Wallis tests were conducted to examine the methylation level differences among quantiles in both cryptic species (B cryptic species Chi-square = 287, *P* < 2.2e^−16^, df = 9; Q cryptic species Chi-square = 826, *P* < 2.2e^−16^, df = 9). Pairwise Wilcoxon signed-rank tests also showed statistically significant differences between quantiles for both B and Q cryptic species (Additional file 1: Tables S26). Regardless of the direction of expression, differentially expressed genes were more often hypomethylated than hypermethylated in both B and Q cryptic species, and significant negative linear correlations (B cryptic species Spearman’s rho = −0.62, *P* < 2.2e^−16^; Q cryptic species Spearman’s rho = −0.48, *P* < 2.2e^−16^) were observed between methylation levels and the degree of differential gene expression (Fig. 3b, d).

**Fig. 4:**
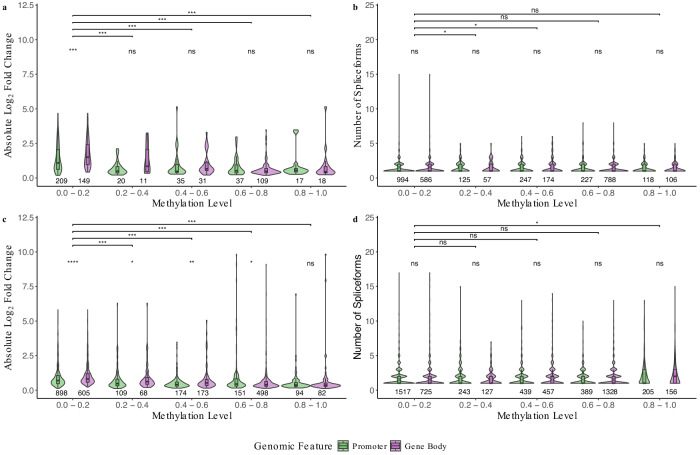
Methylation levels (0.0–0.2, 0.2–0.4, 0.4–0.6, 0.6–0.8, and 0.8–1.0) of promoters and gene bodies in relation to gene expression and alternative splicing following virus acquisition in *Bemisia tabaci.* The B cryptic species methylation levels of promoters and gene bodies corresponding to (**a**) gene expression and (**b**) alternative splicing. The Q cryptic species methylation levels of promoters and gene bodies corresponding to (**c**) gene expression and (**d**) alternative splicing. Counts of genes can be found under their respective boxes. Comparisons between non-viruliferous and viruliferous groups were conducted via Wilcoxon signed-rank tests. Statistical significance levels are indicated by asterisks in the figure: ns (not significant) for *p* > 0.05, * for *p* ≤ 0.05, ** for *p* ≤ 0.01, *** for *p* ≤ 0.001, and **** for *p* ≤ 0.0001.

The association between DNA methylation, gene expression, and AS events (Additional file 8: Tables S27 and S28) was investigated in both whitefly cryptic species. The promoters and gene bodies showed similar methylation patterns in relation to differential expression and in relation to AS events for both cryptic species (Fig. 4; Additional file 1: Table S29). Significant differences between promoter and gene body methylation were found when methylation was below 0.2 in B cryptic species (Fig. 4a) and below 0.8 in Q cryptic species (Fig. 4c). There were no significant differences between promoter and gene body methylation in relation to AS in both cryptic species; however, there was a significant difference between methylation levels 0.2–0.4 and 0.4–0.6 as well as 0.2–0.4 and 0.6–0.8 in B cryptic species (Fig. 4b). Also, there were significant differences between methylation levels 0.2–0.4 and high methylation levels 0.8–1.0 in relation to AS in Q cryptic species (Fig. 4d).

**Fig. 5:**
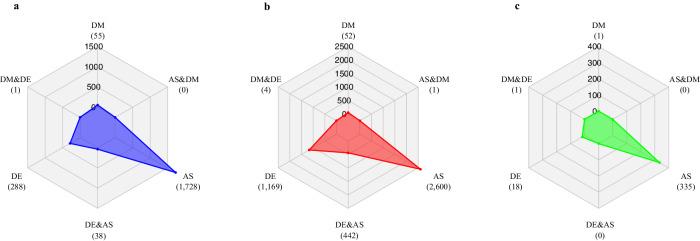
Overlapping differential methylation (DM), differential expression (DE), alternative splicing (AS) events. Similar distributions of overlapping and independent genomic events can be seen in the (**a**) B cryptic species (# of genes = 2503), (**b**) Q cryptic species (# of genes = 5069), and (**c**) one-to-one orthologs (# of genes = 357).

Differential methylation was compared with differential expression and AS events (Additional file 9: Tables S30 and S31). The genes with differential methylation, differential expression, and AS were compared in cryptic species B (Fig. 5a), Q (Fig. 5b), and orthologs between them (Fig. 5c). The analysis revealed that there were low numbers of overlapping genes showing differential methylation and differential expression in both cryptic species, which was found to be insignificant. The overlap of differential methylation and AS in Q also was not found to be significant. When comparing single copy orthologous genes between the two genome assemblies (Additional file 9: Table S32), a high number of AS events was found (Fig. 5c). However, no single copy orthologs were found to be both differentially methylated and alternatively spliced.

### Protein clustering of DMRs and DEGs

**Fig. 6:**
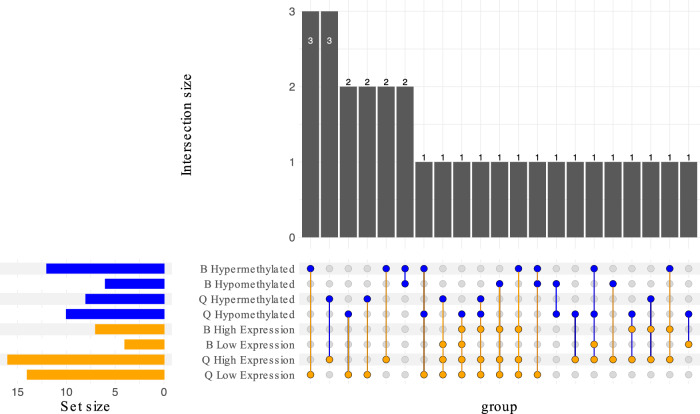
Upset plot (subset includes only groups with at least one methylated set) of gene families within *Bemisia tabaci* B and Q cryptic species relating to the numbers of differentially expressed genes (high and low expression; FDR < 0.05) and differentially methylated genes (hyper- and hypomethylated; *q* < 0.05).

A within- and between-cryptic species gene family approach was taken to characterize the function of genes that may be functionally similar but display divergence in DEG and/or DMR patterns. Five gene families containing 10 genes were found to be exclusive to the B cryptic species, 183 gene families containing 266 genes were found to be exclusive to the Q cryptic species, and 102 gene families containing 483 genes were found in both cryptic species (Fig. 6; Additional file 1: Figs. S5a, S5b, S6). This approach provided a comprehensive view of how genes with similar functions are collectively regulated in response to virus acquisition. By examining the Gene Ontology (GO) terms linked with gene families displaying varying methylation and expression patterns (Additional file 9: Table S33), the functional roles of these proteins within the hierarchical GO categorization were determined. Protein clusters (gene families) meeting the minimum inclusion criteria, being that the gene family contained at least one differentially methylated gene from B and/or Q cryptic species, were explored further for possible associations with virus acquisition.

The most frequent GO terms, associated with clusters meeting the minimum inclusion criteria were investigated, as they may be related to virus acquisiton. These GO terms were: “DNA integration” (GO:0015074; *n* = 3 clusters) and “transposition, DNA-mediated” (GO:0006313; *n* = 2 clusters). Clusters 7, 163, and 172 were characterized as being associated with the GO term “DNA integration” (GO:0015074). However, begomoviruses such as TYLCV are not expected to integrate into vector or host genomes^57^. Clusters 18 and 72 were characterized as being associated with the GO term “DNA-mediated transposition” (GO:0006313), which relates to the movement of transposable elements from one part of the genome to another^58^. The observed changes in methylation and gene expression in clusters 18 and 72 suggest their potential roles in modulating transposon activity during virus acquisition.

Additional protein clusters, meeting the minimum inclusion criteria, with unique GO terms were investigated. Cluster 31 was characterized as a Biological Process (BP) associated with the GO term “translation” (GO:0006412), suggesting its involvement in protein synthesis, which may relate to virus acquisition^59^. Cluster 71 was characterized as a BP associated with the GO term “regulation of protein tyrosine kinase activity” (GO:0061097), which is involved in modulating the activity of tyrosine kinase and indirectly relates to antiviral activity^60–62^. The observed changes in gene expression and DNA methylation in clusters 31 and 71 point to their potential roles in influencing cellular signaling pathways and regulating protein production during virus acquisition, respectively.

Cluster 168 was characterized as a BP associated with the GO term “protein localization to nuclear pore” (GO:0090204), indicating its involvement in mediating protein transport to the nuclear pore complex^63^. The observed changes in DNA methylation and gene expression in cluster 168 suggest its potential role in regulating nuclear import and export during virus acquisition. Cluster 170 was characterized as a BP associated with the GO term “ubiquitin-dependent ERAD pathway” (GO:0030433), suggesting its potential involvement in ER-associated protein degradation via the ubiquitin-proteasome system^64^. The observed changes in DNA methylation and gene expression in cluster 170 indicate its potential role in regulating protein degradation following virus acquisition. Cluster 171 was characterized as a BP associated with the GO term “spliceosomal snRNP assembly” (GO:0000387), indicating its involvement in the assembly of spliceosomal small nuclear ribonucleoproteins^65^.

## Discussion

Epigenetic regulation is known to shape the genetic landscape of organisms at varying intensities. This study examined the influence of DNA methylation on two cryptic species (B and Q) of *B. tabaci*^36,37^. These two cryptic species are of particular interest due to their differences in biological traits, behavior, xenobiotics’ detoxification (including insecticides used in agricultural landscape), and virus transmission abilities^31,32^. Both B and Q cryptic species of *B. tabaci* exist in the Southeastern United States and have previously been shown to be efficient transmitters of the monopartite ssDNA virus –TYLCV^24,44^. Closely related species can exhibit distinct DNA methylation patterns, such as the observed differences between species within genus *Triportheus* (*Characiformes* fishes)^66^. However, intraspecific differences, such as those in the *B. tabaci* cryptic species complex, have not been widely investigated.

Previous studies have shown that insects exhibit significantly lower levels of DNA methylation compared with mammals, and the distribution of DNA methylation across the genome also is variable^67^. Low genomic DNA methylation in *B. tabaci* was expected based on other previous studies^14,68^. Genes frequently subjected to methylation tend be more highly conserved across invertebrate species, indicating slower evolutionary rates and stronger selective constraint^69^. Hypermethylated regions tend to be found within proximity to more highly expressed genes such as housekeeping genes^70–74^. In this study, promoter and gene body methylation levels were compared between B and Q cryptic species. The methylation levels were observed to have a similar pattern of bimodality within both cryptic species. However, this bimodality of the promoter region is not common among invertebrates^75^, suggesting that *B. tabaci* might exhibit unique patterns of promoter methylation. Bimodal promoter methylation has been studied extensively in vertebrate systems, where genes with high DNA methylation levels have been found to be more conserved than those with low DNA methylation levels^76,77^. The methylation of promoters in mammalian systems may serve to reduce the activity of TEs and repress transcription activity^78^. High DNA methylation in promoter regions of *B. tabaci* may be related to stress responses or other functionally significant events, similar to the promoter DNA methylation patterns observed in *Ciona savignyi* (Herdman)^79^. In *B. tabaci*, promoters that were lowly methylated were generally associated with metabolism, signaling, structural, and transport proteins. It also is worth acknowledging that promoter regions’ annotations are dependent on completeness and accuracy of their respective gene body annotations.

Low methylation levels also were observed across most TE superfamilies. However, *copia* retrotransposons, one of the largest TE superfamilies in eukaryotes^80,81^, were found to be targeted more by DNA methylation than other TE superfamilies. Sequence homology between *copia* retrotransposons and the promoter regions of heat shock proteins (HSPs) may indicate that this class of TEs could contribute towards whitefly’s response to biotic stressors such as pathogens^82,83^. HSPs, broadly, are involved in homeostasis and immune response to phytoviruses in several insects including *B. tabaci*^84^. While there was a low number of *copia* retrotransposons found within the genome assemblies of both cryptic species, these TEs also have been observed to be preferentially methylated and accumulate in regions of high CpG density in other arthropod species^50,51^. Members of another major TE superfamily found in eukaryotes, known as *gypsy* retrotransposons^80,81^, were also found to be likely targets of DNA methylation compared with other TE superfamilies in *B. tabaci*. *Gypsy* retrotransposons activation has been found to be involved in *Drosophila* development and immune responses^85^. DNA methylation patterns also were altered in other TE types following virus acquisition within the following TE superfamilies: CACTA TIR transposons, hAT TIR transposons, Mutator TIR transposons, and LTR retrotransposons.

The relationship between DNA methylation and gene expression can vary among different insect species and can be context dependent^1^. Previously reported transcript-derived expression differences in response to virus acquisition in B and Q cryptic species of *B. tabaci*^45^ were compared to the methylation events. The aim was to uncover key methylation patterns affecting virus acquisition and subsequent inoculation in both *B. tabaci* cryptic species. Gene expression levels could potentially be influenced by DNA methylation; however, differential promoter methylation was not found to be associated with gene expression differences in either cryptic species in the current study.

Between the two cryptic species, two pairs of orthologs were identified where members from B and Q each exhibited DMRs, whereas 55 and 56 genes exhibited DMRs in only B or Q cryptic species, respectively. In the B cryptic species, genes with DMRs such as Bta00119 (Tyrosine-protein kinase) suggests potential immune signaling pathways in response to virus acquisition^86^. In the Q cryptic species, genes with DMRs such as BTA018500.1 (40 S ribosomal protein S28) and may relate to regulation of apoptosis following virus acquisition^87^. The first pair of orthologs with DMRs were: Bta03757 (Brinker) in B cryptic species and BTA018959.1 (unnamed protein product) in Q cryptic species. The second pair that had genes with DMRs and were also found to be DEGs *viz*., Bta06672 (Threonine--tRNA ligase) in B cryptic species and BTA026677.3 (39 S ribosomal protein L39, mitochondrial) in Q cryptic species. Threonine--tRNA ligase, which plays an important role in protein biosynthesis, has been previously identified as a candidate for pest control via RNA interference (RNAi) in three rice planthopper species^88^. These distinct genes could indicate differences in biological and physiological responses of the two cryptic species. Additionally, differential methylation may be weakly related to the presence of AS events in *B. tabaci*.

The integration of gene expression analysis with the identification of specific protein clusters associated with GO terms provided valuable insights into *B. tabaci*-virus interactions. Protein clusters (gene families) composed of similar genes and enriched in GO terms such as “DNA integration”, “transposition, DNA-mediated”, “regulation of protein tyrosine kinase activity”, “protein localization to nuclear pore”, “ubiquitin-dependent ERAD pathway”, and “spliceosomal snRNP assembly” are of particular interest mainly because of their relevance to phytovirus acquisition and retention.

The low number of genes with DMRs were expected by random chance, suggesting a lack of conservation of site-specific DNA methylation. As this work delves into the epigenetic regulation of gene expression in *B. tabaci* and its modulation by virus acquisition, it contributes to a broader understanding of insect-vector interactions and the molecular basis of vector-borne pathogen dynamics. This study provides an overview of baseline DNA methylation levels in *B. tabaci* B and Q cryptic species, as well as shows that non-propagative viruses may have minimal, but differential, impact on the global or targeted methylation patterns of their insect vector. It also may be of interest to look at temporal differences in DNA methylation following acquisition of other persistently transmitted viruses in the two cryptic species. Beyond DNA methylation, the study of other forms of epigenetic alterations in *B. tabaci*, such as histone modifications and non-coding RNAs (ncRNAs), may further elucidate the intricate epigenetic regulation underlying their response to virus acquisition.

## Methods

### *Bemisia tabaci* rearing and TYLCV acquisition

Both B and Q cryptic species were reared in separate greenhouses on non-infected tomato, *Solanum lycopersicum* cv. Florida 47 (Seminis Vegetable Seeds, St. Louis, MO, USA) plants, for two generations according to protocols outlined in ref. ^48^. Newly emerged adults were collected and released on TYLCV-infected and non-infected tomato plants for a 72-h acquisition access period (AAP). TYLCV was maintained on tomato plants via *B. tabaci*-mediated inoculation. After 72 h, specimens were collected by removal of leaf tissue. The excised tissues were dipped into a falcon tube to release the whiteflies. The collected specimens were frozen and stored at −20 °C. This set up was replicated five times per each treatment (non-viruliferous and virulifeorus) for both B and Q cryptic species. Single viruliferous adults tested via endpoint PCR has revealed that 90–100% were positive for TYLCV following acquisition^45,89^.

### DNA extraction and confirmation of TYLCV acquisition

Total DNA was extracted from 30 females from each replicate and homogenized using Cetyltrimethylammonium bromide^90^. The B and Q cryptic species and virus acquisition status was confirmed by use of PCR. The specific cryptic species was confirmed with the use of BEM23 primers at an annealing temperature of 55 °C—B cryptic species = 200 bp and Q cryptic species = 400 bp^91^. The presence of TYLCV was confirmed with V2 primers, which encode for the pre-coat protein^92^. Quality control was performed with the use of NanoDrop® Microvolume Spectrophotometer and Qubit® Fluorometer (ThermoFisher Scientific, Waltham, MA, USA).

### Library preparation and sequencing

The extracted DNA was sent to Novogene for bisulfite conversion sequencing. Upon amplification, those sites were read as thiamine and were sequenced with an Illumina HiSeq X to produce short read data. The quality of the raw sequence data was checked with FastQC v0.11.9 (https://www.bioinformatics.babraham.ac.uk/projects/fastqc/) on a per sample basis. Sequencing information derived from FastQC for all samples was organized and visualized using the multiQC v1.11 tool^93^.

### Sequence data processing

Adapter contamination of raw reads was removed via Trimmomatic v0.39^94^ with the following parameters: PE, -phred33, ILLUMINACLIP: NexteraPE-PE.fa:2:30:10 LEADING:3 TRAILING:3 SLIDINGWINDOW:4:15 MINLEN:36. Following the trimming step, the sequence quality was checked again with FastQC. Sequencing information from all samples was organized and visualized using multiQC^93^. This was done to ensure that the adapters have been removed and that only high-quality reads remained. The overall bioinformatics pipeline is shown in Additional file 1: Fig. S7, where the first steps are quality control and trimming of the raw reads. Genome quality was checked with the use of BUSCO v4.0.6 using the Insecta odb10 lineage^54^. The lineage to measure the quality of *B. tabaci* genomes was Insecta odb10, which is a set of 1367 core genes from over 70 insect species and eight insect orders.

High quality trimmed reads were mapped to the respective genomes from *B. tabaci* B and Q cryptic species with the use of Bowtie2 v2.4.1^95^, which was built into Bismarck v0.23.0^96^. The read alignments can be found in Additional file 1: Tables S34 and S35. With bisulfite-converted reads, unmethylated cytosines were converted to uracil. When mapping the reads, methylated cytosines could only map to the reference cytosines, but improper mapping due to single nucleotide polymorphisms were considered by Bowtie2. Cytosines were considered methylated if they were above the methylation difference threshold of five. Thiamine sites in bisulfite-converted reads can align to the reference cytosines; however, the reverse is not true, this mapping asymmetry provided the basis for determining methylated cytosine pileup^97^. The set of raw reads was deduplicated to eliminate the potential PCR contamination.

### Methylation status

As a part of the bioinformatics pipeline, the amount of cytosine in CpG, CHG, and CHH context was determined by Bismarck. Investigation of differential methylation in various genomics regions of *B. tabaci* genome was conducted. The read alignments can be found in Additional file 1: Tables S36 and S37. The R v4.1.0 (https://www.r-project.org/contributors.html) tool to determine differential methylation was methylKit^98^, that implements the Dispersion Shrinkage for Sequencing (DSS) methylation calling software^99^ for determining regions with methylated sites. A sliding window approach was used, as detection of a signal from any individual site was too weak. The Bismark reports were filtered with the methylKit function filterByCoverage() using the parameters: lo.count = 10, lo.perc = NULL, hi.count = NULL, hi.perc = 99.9, pipeline = “bismarkCytosineReport”. The filtered Bismark reports were tiled with the methylKit function tileMethylCounts() using the following parameters: win.size = 1000, step.size = 1000, cov.bases = 5, mc.cores = 24, pipeline = “bismarkCytosineReport”. A comparison of the mapped reads from non-viruliferous and viruliferous *B. tabaci* adults of both cryptic species was generated. The threshold for methylation differential and the qvalue was 10 and 0.05, respectively. Using custom R script (found in file “Figure_3.R” in the Dryad repository 10.5061/dryad.jdfn2z3jn), genes were called if they were within 1.5 kb of a methylated region. SeqMonk (https://www.bioinformatics.babraham.ac.uk/projects/seqmonk/) was used to view the methylation patterns 5 kb upstream and downstream of within both the B and Q cryptic species genomes as well as within various genomic features. The CpG observed/expected (CpGo/e), which serves as an approximation for the number of cytosines in CpG context derived from the evolutionary buildup of methylated cytosines was determined for coding sequences using Notos^100^. Additionally, transposable element determination was conducted with Extensive *De Novo* Annotator (EDTA) v2.1.0^101,102^.

### Transcript expression and alternative splicing events

Previously published RNA sequence data relating to TYLCV-acquisition^45^ were used for downstream analysis. Associated RNA extraction protocols and sequencing methods can be found in ref. ^45^. Adapter contamination of raw reads was removed via Trimmomatic v0.39^94^ with the following parameters: PE, -phred33, ILLUMINACLIP: NexteraPE-PE.fa:2:30:10 LEADING:3 TRAILING:3 SLIDINGWINDOW:4:15 MINLEN:36. Trimmed reads were mapped to the respective B and Q cryptic species genomes by using the program Spliced Transcripts Alignment to Reference (STAR) v2.7.10a^103^ with the following parameters: --outFilterType BySJout --outSAMattributes NH HI AS NM MD --outFilterMultimapNmax 20 --outFilterMismatchNmax 999 --outFilterMismatchNoverLmax 0.04 --alignIntronMin 20 --alignIntronMax 1000000 --alignMatesGapMax 1000000 --alignSJoverhangMin 8 --alignSJDBoverhangMin 1 --sjdbScore 1. Mapped read counts were quantitated with the use of RNA-Seq by Expectation Maximization (RSEM) v1.3.3^104^. DESeq2 was used to determine the expression values of the mapped reads, with a false discovery rate (FDR) < 0.05^105^. The differential exon usage of STAR mapped reads were determined by using DEXSeq^106^. The genomic format files were processed using Another Gtf/Gff Analysis Toolkit (AGAT) v0.8.1 (https://zenodo.org/record/7950165).

### GO-Term analysis of DMR and DEG protein clusters

Gene family orthologs between methylated genes and differentially expressed genes were determined using OrthoVenn2^107^ and OrthoFinder2 v2.5.2^108–110^ with setting -M msa to specify the usage of the multiple alignment using fast Fourier transformation (MAFFT) tool^111^. Alignment statistics were determined with Clustal Omega^112^ and the European Molecular Biology Open Software Suite (EMBOSS) infoalign^113^. The gene families were visualized using the R package ComplexHeatmap v3.17^114^.

### Additional statistical analyses

Statistical analyses were conducted using R v4.1.0, employing the following tests as appropriate: the Kolmogorov-Smirnov test to assess normality or skewness of the data, the Kruskal-Wallis test for non-parametric comparisons among multiple independent groups, the Wilcoxon signed-rank test for non-parametric comparisons between two means within a single group, and Spearman’s rank correlation for assessing monotonic relationships.

### LLM technology implementation and assistance

During the preparation of this work, the authors minimally used ChatGPT 3.5 for assistance with coding and editing. After using this tool/service, the authors reviewed and edited the content as needed and take full responsibility for the content of the publication.

## Data Availability

The bisulfite sequence data generated in this study have been submitted to the National Center for Biotechnology Information (NCBI; https://www.ncbi.nlm.nih.gov/). The data can be accessed under the BioProject accession number PRJNA813453, with BioSamples accession numbers SAMN26503470, SAMN26503469, SAMN26503468, and SAMN26503371. The corresponding Sequence Read Archive (SRA) accession numbers are SRR18266360 through SRR18266379. Supplementary information, additional files, and software can be found in the Dryad repository 10.5061/dryad.jdfn2z3jn. Additional file 1: Tables S1–S37, Figs. S1–S7. Respective tables can be found in Additional file 2: Tables S1–S6; Additional file 3: Tables S7–S12; Additional file 4: Tables S15 and S16; Additional file 5: Tables S18 and S19; Additional file 6: Tables S20–S23; Additional file 7: Tables S24 and S25; Additional file 8: Tables S27 and S28; Additional file 9: Tables S30–S33.
